# Ineffective off-label use of recombinant activated factor VII in a case of bone-marrow transplantation-related gastrointestinal bleeding

**DOI:** 10.1186/1477-9560-4-1

**Published:** 2006-01-18

**Authors:** P Eller, C Pechlaner, CJ Wiedermann

**Affiliations:** 1 Division of General Internal Medicine, Department of Medicine, Medical University of Innsbruck, Anichstrasse 35, A-6020 Innsbruck, Austria; 2 2^nd ^Division of Internal Medicine, Department of Medicine, Central Hospital of Bolzano, Lorenz Boehler Street 5, I-39100 Bolzano (BZ), Italy

## Abstract

**Background:**

For patients with a normal coagulation system, who experience serious bleeding, sound evidence for recombinant activated factor VII (rFVIIa) as an effective haemostatic agent is only scarcely available so far from controlled clinical trials. In systematic reviews on the clinical use of rFVIIa, treatment failures were only rarely reported.

**Case presentation:**

We present a 45-year old, Caucasian male with persistent intestinal bleeding due to enterocolitis associated with cytomegalovirus infection and acute graft-versus-host-disease. He had received allogeneic peripheral blood stem cell transplantation from an unrelated HLA-identical donor because of chronic myelogenous leukaemia diagnosed two years earlier. Bleeding started at day 18 after transplantation with bloody diarrhea, which was treated with multiple transfusions of fresh frozen plasma, platelet, and red blood cell concentrates, and continued relentlessly, despite all efforts, including continued transfusions, high-dose prednisolone, broad antibiotic and antiviral coverage, and tranexamic acid. Recombinant FVIIa was started at boluses of 90–120 μg/kg every 4–8 hours. Despite more than 10 doses, recurrent severe bleeding progressed to refractory shock, multiorgan failure and death.

**Conclusions:**

Little can be concluded from single case reports of clinical improvement, because publication bias in favour of positive effects is likely. Our case suggests that rFVIIa is not a panacea, in particular for severe bleeding after bone-marrow transplantation. As long as rigorous, controlled studies or comprehensive registries are lacking, conventional interventions remain the standard of care in non-haemophilic patients with severe bleeding.

## Introduction

Recombinant activated factor VII (rFVIIa) is an effective haemostatic agent in approximately 90% of patients with hemophilia and inhibiting antibodies, and in other types of complex coagulation disorders [1,2]. It has also been used in patients with a normal coagulation system, who experience serious bleeding. For these other patients, sound evidence from controlled clinical trials is only scarcely available so far. According to a recent systematic review, rFVIIa appears to be relatively safe with a 1–2% incidence of thrombotic complications based on published trials [3]. The main safety outcome in a recent trial on the use of rFVIIa in cerebral haemorrhage was severe thromboembolism at 90 days [4]; severe arterial and venous thromboembolic adverse events were more than three times as common in the rFVIIa groups as in the placebo group (7 percent vs. 2 percent), including seven myocardial infarctions and nine cerebral infarctions (for a combined rate of 5 percent). In the placebo group of this study, no arterial thromboembolic events were observed. In patients with fulminant hepatic failure, rFVIIa was used after conventional means for treating the associated coagulopathy had failed [5]; in these patients, rFVIIa corrected the coagulation defect, but thrombotic complications occurred in two of the four patients (myocardial ischemia and portal vein thrombosis). This indicates that the rate of thrombotic complications depends on disease and patient selection.

Because of this association with serious adverse effects and the substantial cost, off-label use of rFVIIa may only be justified in cases with at least some evidence of benefit and otherwise untreatable life-threatening bleeding. This is particularly true for diseases with unknown efficacy of rFVIIa. In a recent systematic review on the clinical use of rFVIIa from a total of 124 case series and 176 case reports [3], treatment failures were only rarely reported. This may indicate outstanding efficacy of rFVIIa, as an "universal haemostatic agent". Another likely explanation is publication bias. Therefore, we would like to report a patient with life-threatening bleeding that failed to respond to repeated doses of rFVIIa.

## Case

We present a patient with persistent intestinal bleeding due to enterocolitis associated with cytomegalovirus infection and acute graft-versus-host-disease (GvHD). The 45-year old, Caucasian male had chronic myelogenous leukemia (CML) which had been diagnosed 2 years earlier. The CML was in its first chronic phase, it was bcr/abl PCR positive, and had shown only minimal cytogenetic response to conventional treatment with high-dose hydroxycarbamide and interferon alpha. Therefore, he received allogeneic peripheral blood stem cell transplantation from an unrelated HLA-identical donor after conditioning with busulfan and high-dose cyclophosphamide. The leukocyte nadir was on day +5. Graft take was apparent on day +9 after transplantation, with total leukocytes recovering to over 1 G/L. Prophylaxis for GvHD consisted of ciclosporin A, methotrexate, and a short course of low-dose rabbit antithymocyte globulin. In spite of these intensive efforts, acute GvHD of the skin (grade III) developed on day +8, as shown by skin biopsy. Cytomegalovirus infection was diagnosed on day +21 (pp65 was found in 13 of 200,000 leukocytes), and treated with ganciclovir and foscarnet. Acute respiratory failure required respiratory support. The patient was intubated and transferred to the intensive care unit on day +20.

Bleeding started at day +18 after transplantation with bloody diarrhea, which was treated with multiple transfusions of fresh frozen plasma, platelet, and red blood cell concentrates. Endoscopy revealed diffuse intestinal mucosal bleeding, from the stomach to the rectum; biopsy revealed high-grade acute GvHD. Bloody diarrhea continued despite all efforts, which included frequent transfusions of platelet and red blood cell concentrates (Figure), high-dose prednisolone, broad antibiotic and antiviral coverage, and daily tranexamic acid.

Fresh frozen plasma had been administered repeatedly earlier than day +24. After admission to the ICU (day +20) estimated volume losses were replaced by albumin, crystalloids and red blood cell concentrates as long as global coagulation tests were in the high normal range (Figure). All routine coagulation tests (prothrombin time, activated partial thromboplastin time and fibrinogen, were performed at least thrice daily. Fibrinogen never fell below 200 mg/dL (reference > 140), prothrombin time (reference > 70%, Figure) and aPTT always were in plain normal range, except in the terminal 24 hours. Therefore, we considered fibrinogen concentrates not to be useful. We preferred fresh frozen plasma over prothrombin complex concentrates to replace losses. Prothrombin complex concentrates were not used in our patient. Thrombopenia was treated by platelet concentrate transfusion; additional desmopressin was not used.

Renal function remained normal. Pulmonary bilateral infiltrates and need of mechanical ventilation were unchanged. Body temperature was fluctuating between 37.0 to 39.2 °Celsius, systolic blood pressure between 100 and 170 mmHg with intermittent vasopressor support. Heart rate was between 110 and 160 /min.

After a period of apparent overall stability (day +24–27) profuse anal bleeding with declining hemoglobin and blood pressure prompted massive red blood cell transfusions (Figure) and use of vasopressors. Blood coagulation tests remained normal.

rFVIIa was started at day +29 with desperation at the unremitting gastrointestinal bleeding, as compassionate use (Figure). Boluses of 90–120 μg/kg were given every 4–8 hours. After nine doses, rectal bleeding rate decreased, and PT somewhat shortened (% increase), albeit always in the normal range (Figure). rFVIIa was discontinued. Less than 24 hours later, on day +33, massive transrectal bleeding recurred. Hemoglobin fell to 5 g/dL and hypotension required a sharp increase of vasopressor doses. Recombinant FVIIa was started again. Repeat endoscopy confirmed diffuse bleeding from severely lacerated mucosa of stomach and duodenum.

Despite massive transfusions and maximal intensive care, the patient rapidly progressed to multiorgan failure with progression of bilateral pulmonary infiltrates ("white lungs"), anuria, severe acidosis, and vasopressor-refractory hypotension. The patient died one day after start of the second rFVIIa treatment series, on day +34 after transplantation.

## Discussion

rFVIIa was developed for the treatment of hemorrhagic episodes in haemophilic patients with inhibitors to factors VIII and IX. Since its introduction, it has also been used "off-label" to enhance haemostasis in non-haemophilic patients who experience bleeding episodes unresponsive to conventional therapy. Conclusive evidence of its effectiveness in non-haemophilic conditions from controlled clinical trials is not yet available [6,7]. "Last-ditch" use of rFVIIa in patients with massive haemorrhage resistant to conventional treatment did not rescue these patients or significantly alter outcomes in a retrospective study by Clark et al [8]. None of the published randomized, placebo-controlled, double-blind clinical trials of rFVIIa in non-haemophilic patients with severe bleeding found significant differences in clinical outcomes. Prophylactic rFVIIa dosing did neither significantly reduce the number of blood products transfused after major partial hepatectomy [9], nor did it decease the perioperative blood loss in reconstruction surgery for traumatic fracture of pelvis and acetabulum [10]. Furthermore, there was no significant differences in mortality in patients with cirrhosis and upper gastrointestinal bleeding when treated with rFVIIa or placebo [11]. Results from these prospective randomized placebo-controlled trials on the use of rFVIIa as an adjunct for prevention and therapy of bleeding in surgery and liver diseases recommended, thus, conventional intervention for prevention and control of haemorrhage in non-haemophilic patients and contrast thereby to other more optimistic studies [3-5].

Severe bleeding refractory to standard support is also common in patients undergoing bone marrow transplantation. In 2001, Blatt and co-workers reviewed their experience with rFVIIa in three patients treated for pulmonary haemorrhage, hemorrhagic cystitis, and gastrointestinal bleeding [12]. Transient clinical responses in gross haematuria and in pulmonary haemorrhage were noted within several days of starting rFVIIa, but bleeding in new sites and renewed bleeding at the initial site prompted discontinuation of the drug. From that case series it was concluded that large randomized studies are needed before definitive off-label use in the setting of bone marrow transplantation can be recommended. On the other hand, successful treatment with rFVIIa of two bone marrow transplant patients with pulmonary haemorrhage and cyclophosphamide-induced hemorrhagic cystitis was reported, respectively [13,14]. In the recent, randomised study of rFVIIa in 100 patients with bone marrow transplant-related bleeding [15], the primary outcome, a bleeding score, was not different between rFVIIa and placebo.

Our case illustrates that rFVIIa is not a panacea for refractory bleeding, in particular for severe bleeding after bone-marrow transplantation, and in particular for gastrointestinal bleeding. Bleeding is the extravasation of blood. Extravasation of blood is the inevitable consequence of injury to vascularized tissues. The extent and duration of extravasation depends not only on blood coagulation (platelet aggregation and fibrin formation), but to an often underappreciated degree also on the degree of damage to the vessel wall (how big are the holes in the vessel wall), the pressure gradient between vessel lumen and tissue (i.e. blood pressure), and finally on whether compression at the site on injury is possible (by the hematoma itself or by medical intervention). Most gastrointestinal bleedings occur in individuals with normal blood coagulation, e.g. through arrosion of vessels by ulcers, or by Mallory-Weiss tears. In the gastrointestinal lumen, diffuse mucosal, and vascular, damage cannot usually be controlled by compression. Therefore, the expectation that diffuse mucosal gastrointestinal bleeding may be controlled by augmenting blood coagulation may be considered naive in many such patients.

Our patient had normal blood coagulation tests, but nevertheless bleeded recurrently. We feel, that with such severe diffuse damage to gastrointestinal mucosa and vessels, as in our patient, interventions at improving blood coagulation are of little benefit. This does not only apply to rFVIIa, but also on other measures available, such as factor concentrates, plasma, antifibrinolytics, desmopression or others. To stop bleeding, it is essential to stop the process of tissue distruction and of vessel damage.

In our patient, we tried rFVIIa, with growing despair among the young family and among us carers, and with some hope based on favorable outcomes with rFVIIa in published case reports. Without continous endoscopy, it is difficult to verify any short-term changes of bleeding rates in gastrointestinal bleeding, due to the intermittent nature of intestinal emptying. The first course of rFVIIa in our patient may have led to some improvement, but the concomitant multiple red blood cell transfusions and steadily declinig hemoglobin thereafter (Figure) warrant caution with any such conclusion. The massive recurrent bleeding only 24 h after the last of nine rFVIIa doses is enough evidence, at least in our view, to judge the intervention ineffective.

Our patient did not exsanguinate (Figure); the last path to his death was rapidly progressing multiorgan failure. Bleeding very likely contributed to the tragic outcome, in particular the terminal, most severe episode.

Little can be concluded from single case reports of clinical improvement, because publication bias in favour of positive effects is likely. As long as rigorous, controlled studies or comprehensive registries do not prove benefit of new treatments, conventional interventions remain the standard of care in non-haemophilic patients with severe bleeding [7].

**Figure 1:**
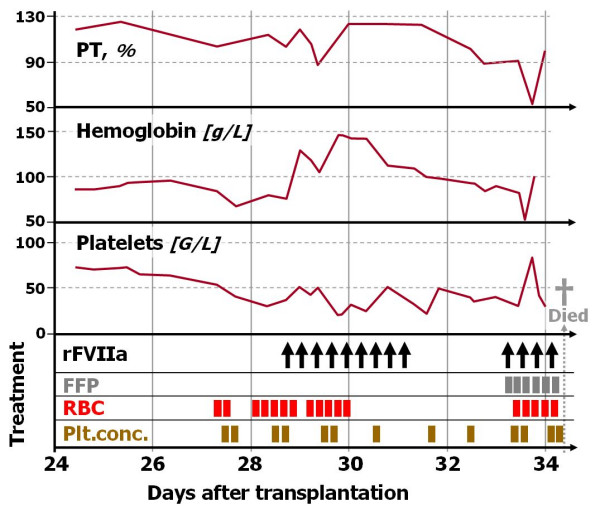
**Time Course**. Time course of prothrombin time (PT), haemoglobin, and platelet count in relation to treatment with rFVIIa and blood components. Arrows symbolize one bolus of rFVIIa, bars one unit of fresh frozen plasma (FFP), red blood cell (RBC), and platelet concentrates (Plt.conc.), respectively.
